# Mineral Montmorillonite Valorization by Developing Ni and Mo–Ni Catalysts for Third-Generation Green Diesel Production

**DOI:** 10.3390/molecules27030643

**Published:** 2022-01-19

**Authors:** Sotiris Lycourghiotis, Eleana Kordouli, John Zafeiropoulos, Christos Kordulis, Kyriakos Bourikas

**Affiliations:** 1 School of Science and Technology, Hellenic Open University, Parodos Aristotelous 18, GR-26335 Patras, Greece; sotirislyk@gmail.com (S.L.); jzafeirop@eap.gr (J.Z.); 2 Department of Civil Engineering, University of Peloponnese, 1 M. Alexandrou Str., GR-26334 Patras, Greece; 3 Department of Chemistry, University of Patras, GR-26504 Patras, Greece; ekordouli@upatras.gr (E.K.); kordulis@upatras.gr (C.K.); 4 Foundation of Research and Technology, Institute of Chemical Engineering Science (FORTH/ICE-HT), Stadiou Str. Platani, P.O. Box 1414, GR-26500 Patras, Greece

**Keywords:** green (renewable) diesel, nickel catalysts, montmorillonite, waste cooking oil, selective deoxygenation

## Abstract

Four Ni catalysts and one Mo–Ni catalyst supported on montmorillonite were synthesized, characterized by various techniques and evaluated, under solvent-free conditions, for the production of green diesel from waste cooking oil. The optimum Ni content was found to be 20 wt.%. The addition of 2 wt.% Mo to the catalyst resulted in a considerable increase in the amount of green diesel hydrocarbons. The Mo species, moreover, led to a decrease in the (C15 + C17)/(C16 + C18) ratio, which is beneficial from the viewpoint of carbon atom economy. The promoting action of Mo was mainly attributed to the synergy between the oxygen vacancies on the surface of the well-dispersed Mo(V) and Mo(VI) oxides and the neighboring Ni^0^ sites. The optimum reaction conditions, for achieving a proportion of liquid product in the green diesel hydrocarbons (C15–18) equal to 96 wt.%, were found to be 350 °C, 3 g of catalyst per 100 mL of waste cooking oil and 13 h reaction time. These conditions correspond to an LHSV of 2.5 h^−1^, a value that is considered quite reliable from the viewpoint of industrial applications. Thus, the cheap and abundant mineral montmorillonite is proved a promising support for developing efficient Ni–Mo catalysts for green diesel production.

## 1. Introduction

Fossil fuels are the primary energy sources for the transportation sector which consumes about 28% of the global energy production [1]. Their consumption is gradually increasing to meet the energy demands of the Earth’s growing population. As the amount of these fuels is not unlimited, their increasing consumption will lead to the progressive depletion of oil reserves during the 21st century [2,3]. In addition, the emission of carbon dioxide from the combustion of fossil fuels is responsible for global warming and thus for climate change [2,3]. In view of the above, the production of transportation fuels from renewable “carbon neutral” resources such as biomass is indispensable for the progressive minimization of the use of fossil fuels. Two types of biomass are currently used for the production of transportation fuels: bioethanol as a substitute for gasoline and biodiesel as a substitute for fossil diesel. The first is produced from sugar and starch by yeast fermentation and the second from the natural triglycerides contained in vegetable oils and animal fats via transesterification with methanol. However, biodiesel (a mixture of fatty acid methyl esters) presents unfavorable cold flow properties and delivers lower fuel mileage compared to fossil diesel. This is due to the presence of oxygen in its ester structure. Therefore, it should be blended with petrol diesel in proportions up to 20% in the usual combustion engines [2]. The second problem encountered when using bioethanol is also due to its oxygen content. As well as these problems, the production of the aforementioned “first-generation biofuels” using sugar, starch and edible vegetable oils competes for land and water with the production of food. The lignocellulosic biomass of plant waste products (consisting of 40–50% cellulose, 25–35% hemi-cellulose, 15–20% lignin and small amounts of other components [4]) is recognized as a promising alternative source of transport fuels for overcoming the aforementioned competition with food production [5]. In spite of an intensive research effort over the last decades, the complex structure of lignocellulosic biomass, mainly that of lignin, has not so far allowed the development of a cost-effective process [6]. However, research continues aimed at developing more efficient catalysts for transforming lignin to hydrocarbons [7]. Triglyceride biomass is attractive because it has a much simpler structure than lignocellulosic biomass. Moreover, the ratio of oxygen to the combustible carbon and hydrogen atoms in a molecule of triglyceride is relatively small. This justifies the intensive research effort over the last decades regarding the transformation of natural triglycerides and related compounds into n-alkanes in the diesel range (green diesel) via hydrotreatment [8,9,10,11]. Green diesel, with a cetane number of 85–99 outperforms petroleum-derived fuels with a cetane number of 45–55, and after a mild hydroisomerization it can be used as stand-alone fuel in conventional diesel engines. Based on the triglyceride feedstock used, green diesel can be classified into four generations (first generation: edible vegetable oils; second generation: non-edible vegetable oils; third generation: residual vegetable oils and animal fats; and fourth generation: algal oils [12]). Although the feedstocks used in most studies are fresh edible vegetable oils and model compounds, there are increasing numbers of studies where non-edible vegetable oils such as jatropha oil are used [12,13,14,15,16,17,18]. More usefully, there are increasing numbers of studies dealing with the utilization of residual fatty raw materials for the production of third-generation green diesel, such as waste cooking oils [19,20,21,22], yellow grease and hemp seed oils [23], brown grease oil [24], distilled fatty acids [24,25,26,27], oil from chicken fat [28,29] and oil extracted from spent coffee grounds [30,31,32,33,34].

**Figure 1:**
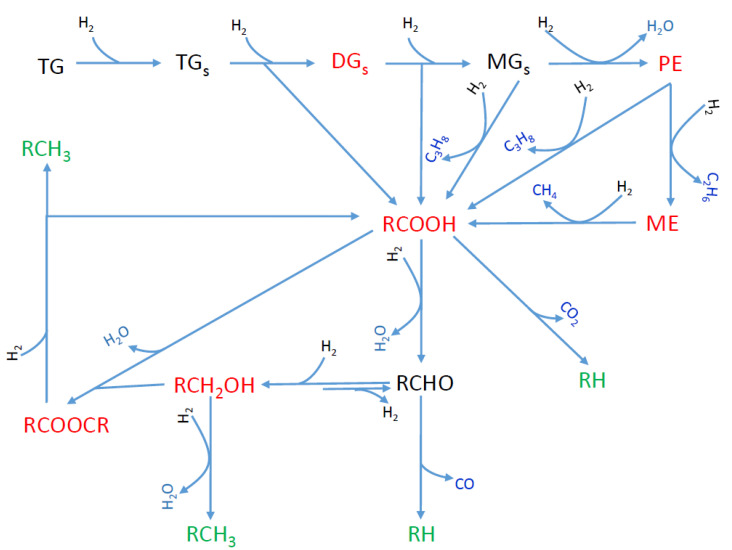
SDO reaction network. TG, TG_S_, DG_S_, MG_S_, PE and ME are the initial triglycerides, the saturated triglycerides, the saturated diglycerides, the saturated monoglycerides, the propyl-esters and the methyl-esters, respectively. The other molecules are represented by their chemical formulas [43]. (Molecules written in green, red and blue are final, intermediate and gas-phase products, respectively).

The production of green diesel by hydrotreatment of natural triglycerides requires oxygen removal without fragmentation of the side chains of triglycerides through so-called selective deoxygenation (SDO). This was initially studied over noble metals, mainly Pd supported on carbon [35,36] and Pt supported on various supports [37,38,39]. Although these catalysts are very efficient, their high price increases the cost of the procedure. The classical Ni–Mo or CoMo/γ-Al_2_O_3_ sulfided catalysts are also efficient for SDO [40,41,42]. Nevertheless, when using these catalysts in a stand-alone process we must add sulfur compounds to the feedstock in order to maintain the stability of the catalyst’s sulfided state and hence its high activity. Obviously, the presence of sulfur in the end product could lower its quality. In view of the above, the research effort was progressively shifted to the cheaper and quite efficient supported nickel catalysts acting in their reduced state. The research development, reviewed up to 2016 by our group [11], continues intensively today. The SDO proceeds through the following reaction network over the metallic nickel nanoparticles (Figure 1) [11,22,43]: the unsaturated side chains of triglycerides are quickly saturated, and this is followed by the rapid progressive decomposition of the O-C bonds on the glycerol backbone side, resulting in di- and mono-glycerides and then propane, releasing one molecule of fatty acid after each decomposition. The fatty acids might then be decarboxylated to n-alkanes and/or hydrogenated/dehydrated to fatty aldehydes, which then are decarbonylated to n-alkanes. Although both pathways lead to the formation of n-alkanes with one carbon atom fewer than that of the triglyceride chain (odd number of carbon atoms), the second pathway is much more probable, prevailing in the whole reaction network over the reduced nickel catalysts. Alternatively, the aldehydes are reduced to fatty alcohols which then are dehydrated, and the produced olefins are hydrogenated to n-alkanes with a number of carbon atoms equal to that of the triglyceride chain (even number of carbon atoms). The intermediate alcohols may react with the intermediate fatty acids leading to long-chain esters. These may undergo SDO, resulting again in n-alkanes.

Although the reduced nickel catalysts are very promising for SDO, they present some drawbacks such as the non-negligible activity regarding C-C hydrogenolysis at moderate temperatures and methanation of the CO produced. Both increase the hydrogen consumption, and the former is also related to a decrease in carbon yield and an increase in carbon deposition (deactivation). Several methodologies have been developed to minimize these problems involving the use of suitable supports, nickel loading, preparation methods and optimized experimental conditions (mainly reaction temperature and hydrogen pressure) [11], as well as modification of the structure of the supported nickel through development of nickel phosphide [44,45,46], nitride [47,48] or carbide catalysts [32,48]. Another very promising prospect for overcoming the above drawbacks and further increasing the efficiency of nickel catalysts is the use of promoters, among which molybdenum oxides are the most effective [20,21,49,50,51,52,53,54,55,56,57,58,59,60,61,62,63,64,65]. The literature survey indicated that excellent promoting action is observed in molybdenum oxides, mainly for catalysts with a Ni/(Ni+Mo) atomic ratio close to 0.85–0.95 [20,21,49,50,52,55,56,59,62,63].

Both the unpromoted and promoted nickel catalysts used to date for green diesel production are usually supported on synthetic supports (γ-alumina, silica, silica–alumina, synthetic zeolites, active carbons, carbon nanotubes, zirconia, titania, ceria, etc. [11,20,21,49,50,51,52,53,54,55,56,57,58,59,60,61,62,63,64]). Studies using cheap minerals as natural supports are very scarce [65,66,67]. Taking into account the fact that larger and larger amounts of catalysts will be required for green diesel production, the use of minerals seems to be attractive for decreasing the catalyst cost in the context of sustainable development and the cyclic economy. This motivated a relevant research program undertaken by our group. In the frame of this program, we have successfully developed nickel catalysts supported on mineral palygorskite and mordenite, for the production of third-generation green diesel [65,66,67].

In the present work, we extend our studies by developing nickel and molybdenum–nickel catalysts supported on mineral montmorillonite for the production of third-generation green diesel. Montmorillonite is the main ingredient of bentonite, a well-known rock [68]. Greece is the second-highest exporter of bentonite in the world, after the United States, with exports exceeding one million tons per year and total feedstocks of 100 million tons.

**Figure 2:**
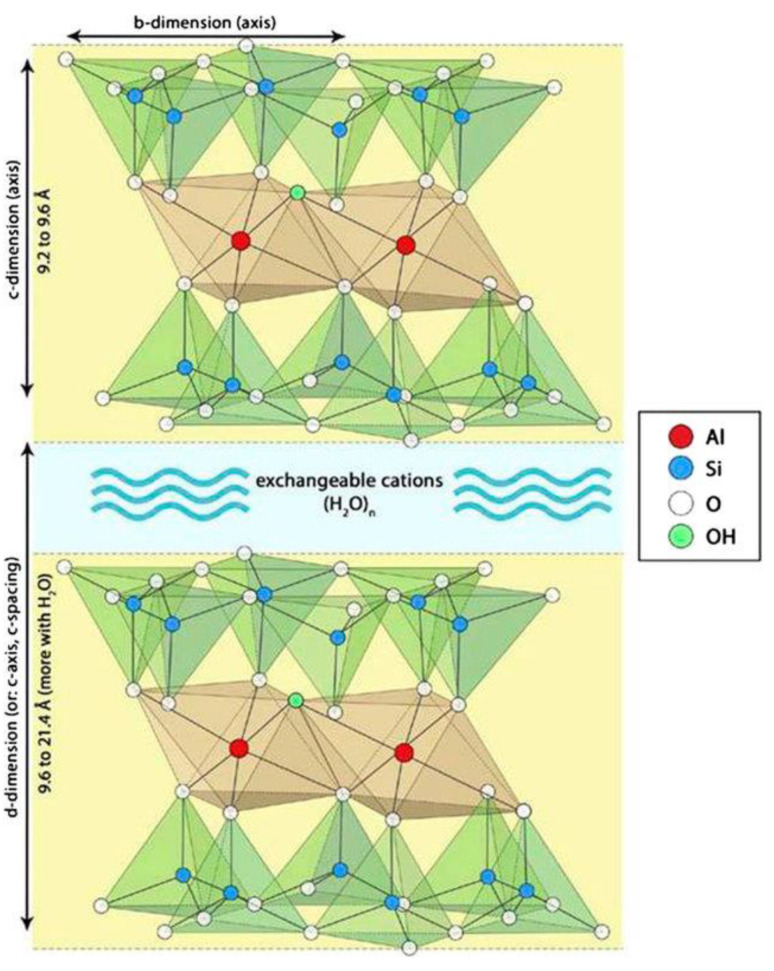
Montmorillonite unit [69]. The triple layers are principally extended along the b-axis, whereas the unit is repeated several times along the c-axis. This creates plate-shaped particles.

The structure of montmorillonite is illustrated in Figure 2. It can be seen that the montmorillonite unit involves two adjacent triple layers (crystals) separated by a region containing exchangeable cations (e.g., Na^+^ or Ca^2+^), together with water molecules [69]. Each triple layer is composed of two layers of tetrahedral SiO_4_ units which are connected through a layer of octahedral AlO_6_ units. Several Al^3+^ ions are replaced by Mg^2+^ and/or Fe^2+^ ions and several Si^4+^ ions are replaced by Al^3+^ ions. Thus, a negative charge is developed which is compensated by the aforementioned exchangeable cations. It is important to note that acid sites are developed on the montmorillonite surface. An improvement in the physicochemical properties of mineral montmorillonite can be achieved via treatment with acidic solutions [70,71,72,73,74]. In a previous study, we investigated the influence of various parameters of the acid treatment of mineral montmorillonite (originating from soils in the Greek islands) on its physicochemical properties and catalytic efficiency in the transformation of limonene into high-added-value isomers and p-cymene [75]. This was done in the context of the development of catalysts/catalytic supports based on mineral mordenite and palygorskite for the production of green diesel [65,66,67] and green products [76,77,78], as well as on montmorillonite for the production of green products by valorizing renewable/waste raw materials [75].

Having utilized mineral montmorillonite successfully for the production of green products by valorizing renewable/waste raw materials [75], it is reasonable to ask whether this very cheap mineral could also be used for the development of supported Ni and Mo–Ni catalysts for the production of third-generation green diesel using waste cooking oil (WCO) as the feedstock. Answering this question is the main goal of the present study. It should be noted that, to the best of our knowledge, no articles have been presented so far in the literature dealing with the production of third-generation green diesel accelerated by nickel catalysts supported on mineral montmorillonite. Another important question is whether the well-known promoting action of molybdenum species in supported nickel catalysts is also valid for nickel catalysts supported on montmorillonite. In order to investigate the aforementioned issues, we adopted the following experimental methodology. First, we studied the effect of nickel loading on the physicochemical characteristics and the catalytic efficiency for the production of third-generation green diesel using waste cooking oil (WCO) as the feedstock. This was realized by preparing, characterizing through various techniques and evaluating nickel catalysts with different loadings, which allowed the most promising loading to be selected. Using this loading, we then synthesized, characterized and evaluated the corresponding molybdenum–nickel catalyst, which proved to be much more efficient than the corresponding unpromoted nickel catalyst. Finally, we evaluated the promoted catalyst under different reaction conditions (temperature, catalyst mass and reaction time) in order to investigate the effect of these conditions on catalytic efficiency. The ultimate goal was the development of an efficient catalyst supported on mineral montmorillonite for the production of third-generation green diesel.

The mineral montmorillonite (MM) treated with hydrogen chloride aqueous solution was denoted MM(H) and the solid produced (following the preparation protocol for supported nickel catalysts but without adding nickel) from this treated montmorillonite was denoted 0NiMM(H). Four monometallic catalysts with different nickel contents in the range 10–40 wt.% Ni were synthesized by deposition–precipitation using the MM(H) as a support, in order to investigate the effect of nickel loading, denoted 10Ni/MM(H), 20Ni/MM(H), 30Ni/MM(H) and 40Ni/MM(H). Finally, one bimetallic catalyst was synthesized by co-deposition–precipitation using the MM(H) as a support, in order to investigate the molybdenum promoting effect, denoted 20Ni 2Mo/MM(H). We note that the Ni/(Ni+Mo) atomic ratio in the bimetallic catalyst was equal to 0.94.

## 2. Results and Discussion

### 2.1. Catalyst Characterization

#### 2.1.1. Texture

The textural characteristics of the supports and the catalysts are given in Table 1. Inspection of this table shows that the greater portion of the ΒΕΤ specific surface area (S_ΒΕΤ_) for the samples MM and MM(H) was due to mesopores (2–50 nm) and macropores (50–100 nm), estimated by the BJH specific surface area (S_BJH_). The microporous specific surface area (S_micro_), determined for pores in the range 0.0–1.7 nm, was smaller.

**Figure 3:**
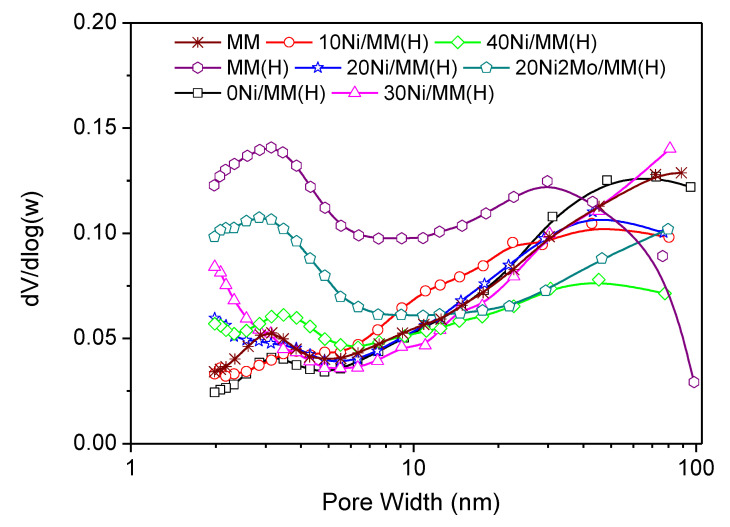
Pore volume distribution of the supports and the catalysts.

This is also visualized in the pore volume distribution curves (Figure 3), which show pores in the range of 2–6 nm centered at about 3.0 nm, accompanied by many pores in the range of 10–100 nm. The mean values of pore diameters (MPD), given in Table 1, are in good agreement with the pore volume distribution curves. The acid treatment drastically increased the S_BJH_ value and thus the value of S_ΒΕΤ_ (Table 1). This is related to the increase in the pore volume in the aforementioned range of 2–6 nm of the small mesopores (Figure 3) and was attributed to the reorganization/dislocation of the triple layers of montmorillonite (Figure 2) inside a plate-shaped particle of this mineral [75]. Heating at 500 °C upon activation of the samples 0Ni/MM(H)–40Ni/MM(H) resulted in reverse reorganization of the triple layers of montmorillonite and a decrease in the pore volume of the small mesopores in the range of 2–6 nm (Figure 3). This was reflected in a decrease in the S_BJH_ value. It may be seen that the presence of nickel species on the support surface decelerates the aforementioned decrease of the pore volume in the range of 2–6 nm (Figure 3) and thus the decrease in the S_BJH_ value (Table 1). For example, S_BJH_ takes the value of 36 m^2^g^−1^ in the sample without nickel, 0Ni/MM(H), and 54 m^2^g^−1^ in the sample with the maximum nickel loading, 40Ni/MM(H). Another significant observation is that the presence of nickel in the samples above 10 wt.% Ni causes an increase in the pore volume in the micropore range, as shown by extrapolating the pore volume distribution curves to micropores (Figure 3).

This is reflected in the impressive increase in the S_micro_ value (Table 1), which is maximized in the sample 30Ni/MM(H) and can be attributed to some kind of pillaring of micropores brought about by the nickel species inserted inside them. Consequently, the aforementioned deceleration of the decrease in the S_BJH_ value and the considerable increase in the S_micro_ value due to the presence of nickel species results in an increase in the BET specific surface area, which is maximized in the sample 30Ni/MM(H) (Table 1). The above effects are more pronounced in the bimetallic MoNi catalyst, 20Ni2Mo/MM(H), which exhibits the highest pore volume in the small mesopore range of 2–6 nm and in pores smaller than 10 nm (Figure 3), and thus the highest values of S_BJH_ and S_BET_ (Table 1) among the catalysts studied. This beneficial role of molybdenum species regarding the texture of the catalysts is in line with the literature [11].

#### 2.1.2. Phase Identification

**Figure 4:**
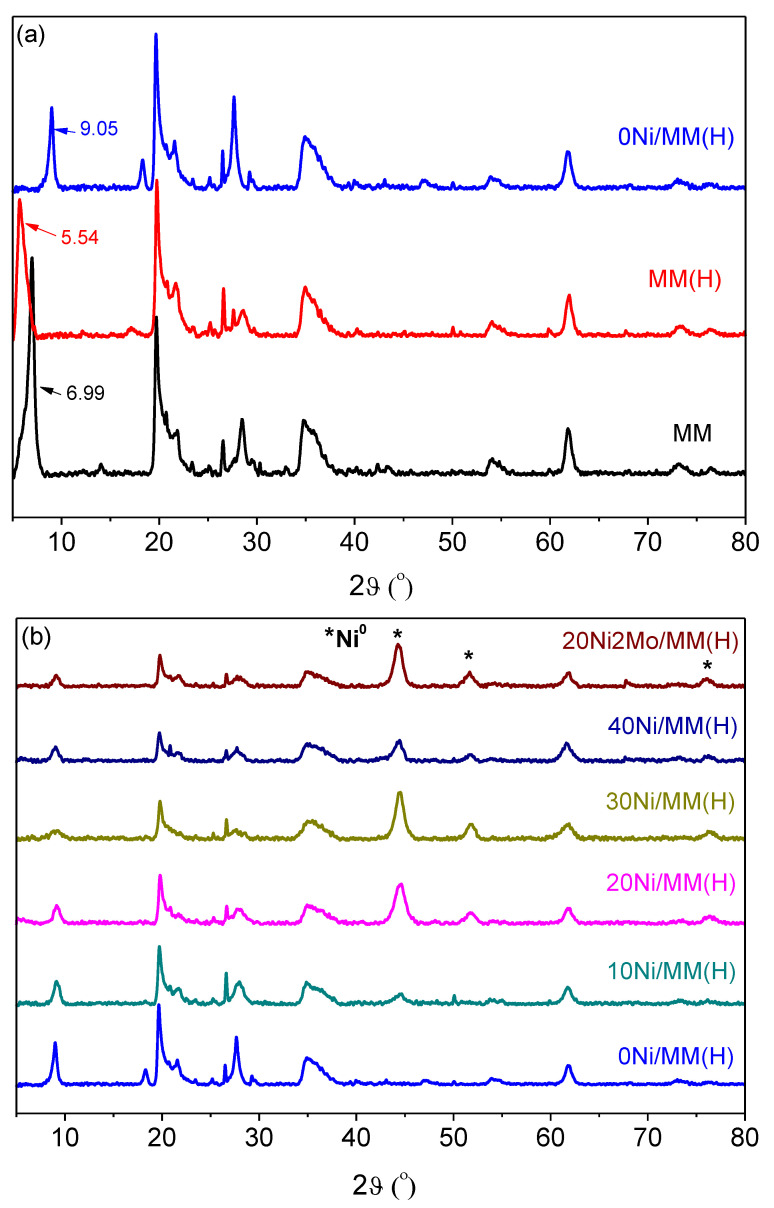
XRD patterns of the samples ((**a**) supports and (**b**) catalysts).

The XRD patterns of the samples studied (Figure 4) show the presence of mineral montmorillonite (2θ values: 6.99, 14.05, 19.69, 20.73, 21.90, 26.49, 28.32, 34.71, 54.01 and 61.74°; JCPDS # 01-073-1490) [75,79,80], as well as the presence of supported nickel nanoparticles (2θ values: 44.5, 51.8 and 76.3°; JCPDS 04-0850) [79,81,82]. The absence of peaks due to the molybdenum species in the promoted sample, 20Ni2Mo/MM(H), strongly suggests the high dispersion of these species.

The application of the Debye–Scherrer equation to the XRD peaks at 2θ < 10° allowed the determination of the mean size of the support nanocrystals. This ranged randomly between 14.2 and 14.4 nm, irrespective of the acid activation and heat treatment of the mineral or the presence of metallic nickel nanoparticles. Moreover, the fact that there was no change in the position of the montmorillonite XRD peaks in the range of 14.05–61.74° shows that the montmorillonite crystal structure inside the triple layers (Figure 2) is not affected by the aforementioned factors. In contrast, the position of the peak at 2θ = 6.99°, corresponding to the (0 0 1) orientation in MM, was decreased to 2θ = 5.54° after the acid treatment (MM(H)). This peak was shifted to 2θ = 9.05° after activation, irrespective of the nickel and molybdenum content. The (0 0 1) orientation corresponds to the c-axis illustrated in Figure 2, while the distance between the crystal planes represents the distance between the down edges of the up and down triple layers, which involves the interlayer region (d-dimension in Figure 2). Therefore, the decrease in the 2θ value after the acid treatment indicates that the replacement of the Na^+^ and Ca^2+^ ions located in the interlayer region by H_2_O/H_3_O^+^ caused an increase in its width. In fact, the length of the d-dimension, as determined by XRD, increased from 1.3 to 1.6 nm due to the acid treatment. In contrast, the increase in the 2θ value after activation indicates a significant decrease in the interlayer width, presumably due to the removal of interlayer water molecules. In fact, the length of the d-dimension decreased from 1.6 to 1 nm due to the activation.

The application of the Debye–Scherrer equation to the XRD peaks at 2θ = 44.5° allowed the determination of the mean size of the supported nickel nanocrystals (MCS_Ni_^0^, Table 1). This size increased with the nickel content. The presence of molybdenum species did not affect the size of the nickel nanocrystals with respect to the corresponding unpromoted sample. Taking into account the mean size of the nickel nanocrystals, we calculated the specific surface area of the exposed nickel (S_Ni_^0^) (active surface). It can be observed that this increased considerably from the sample with 10% Ni to the sample with 20% Ni, and then increased slightly with nickel content. The presence of molybdenum species did not affect this parameter (Table 1).

**Figure 5:**
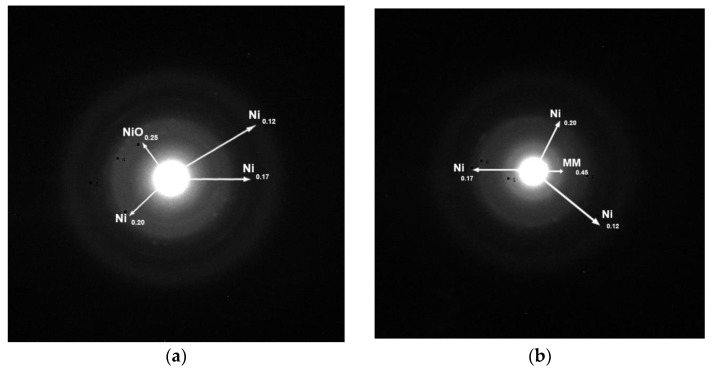
Typical electron diffraction patterns of the samples (**a**) 20Ni/MM(H) and (**b**) 20Ni2Mo/MM(H).

The structure of the catalysts was also investigated by electron diffraction. Two typical electron diffraction patterns are illustrated in Figure 5. The presence of metallic nickel, detected in both samples, is in full agreement with the XRD results. In the unpromoted samples, NiO nanocrystals were also identified, though these were not detectable by XRD presumably due to their very small size. This phase was not identified in the promoted sample. The diffraction at about 0.45 nm, detected in the promoted sample, could be attributed to the (1 0 0) crystal plane of the montmorillonite used as the support (JCPDS # 01-073-1490).

#### 2.1.3. Morphology and Mapping at Nanoscale and Microscale

**Figure 6:**
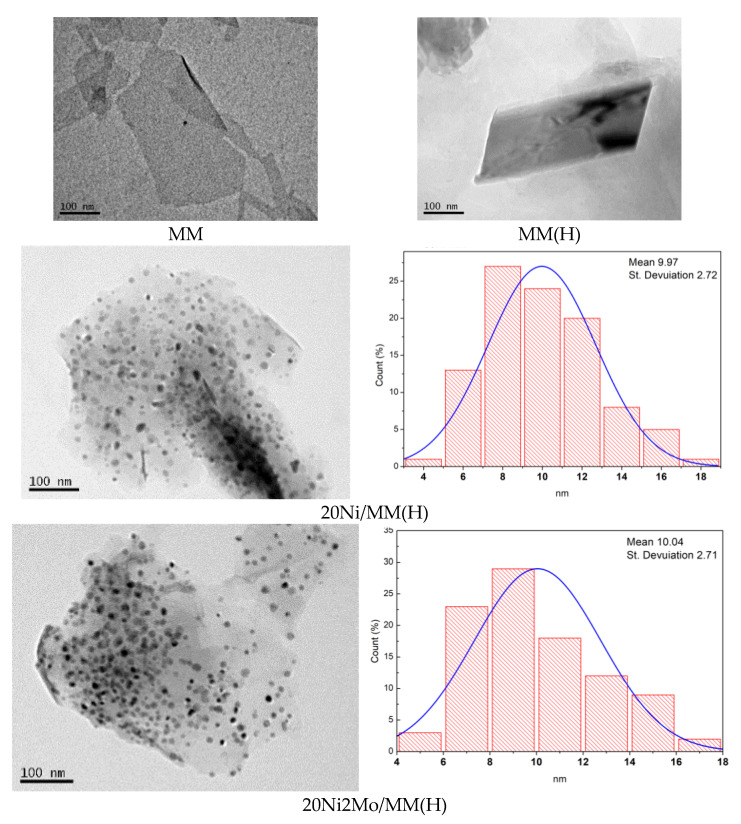
Representative TEM images and the corresponding nickel particle size distributions.

The morphology of the supports and catalysts at the nanoscale was studied using TEM. Representative TEM images are illustrated in Figure 6. It may be seen that the samples MM, MM(H), 20Ni/MM(H) and 20Ni2Mo/MM(H), taken as examples, exhibited a plate-shaped morphology at the nanoscale that was due to sets of montmorillonite units, as depicted in Figure 2. In contrast, the supported nickel nanoparticles exhibited a rather granular morphology. The particle size distributions of granular nanoparticles obtained in the samples 20Ni/MM(H) and 20Ni2Mo/MM(H) are also illustrated in Figure 6. It can be seen that the size of the most of nickel nanoparticles was in the range of 6–12 nm, with a mean value of about 10 nm. This is similar to the mean size of nickel nanocrystals determined by XRD for these samples (Table 1). Moreover, it can be seen that the presence of molybdenum species did not influence the nickel particle size distribution and the mean size of the nickel nanocrystals to any great extent, in full agreement with the XRD results (Table 1).

**Figure 7:**
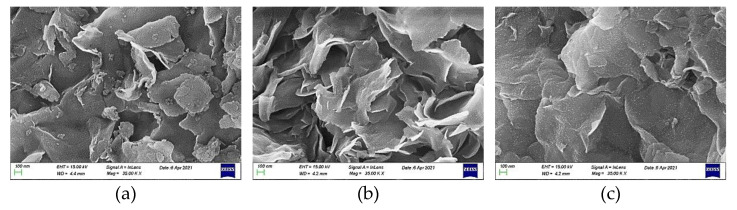
Representative SEM images of the samples (**a**) MM; (**b**) 20Ni/MM(H) and (**c**) 20Ni2Mo/MM(H). (magnification 35.000 KX).

Examining the mineral montmorillonite at the microscale, we observed that agglomerates of the plate-shaped support nanoparticles, as determined by TEM (Figure 6), constituted flower-like assemblies, as shown in the representative SEM images recorded at much lower magnification for the three samples taken as examples (Figure 7). A similar morphology was observed at even smaller magnification.

**Figure 8:**
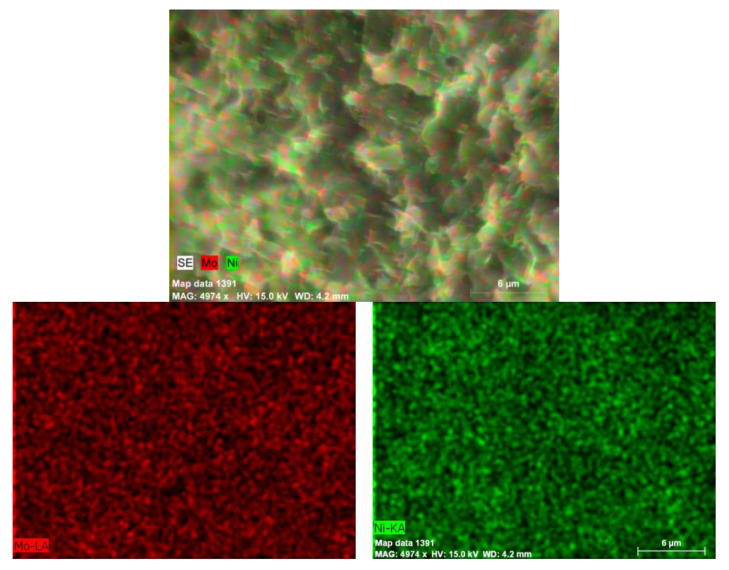
SEM–EDX maps of nickel and molybdenum in the sample 20Ni2Mo/MM(H).

The SEM–EDX mapping of the nickel and molybdenum at the microscale showed a rather uniform distribution for both metals above the support particles. A typical map is shown for the bimetallic sample, 20Ni2Mo/MM(H), in Figure 8. It is important to observe that the nickel and molybdenum species are located very close together, at least at the microscale. This observation is important for interpreting the promoting action of the molybdenum species.

**Figure 9:**
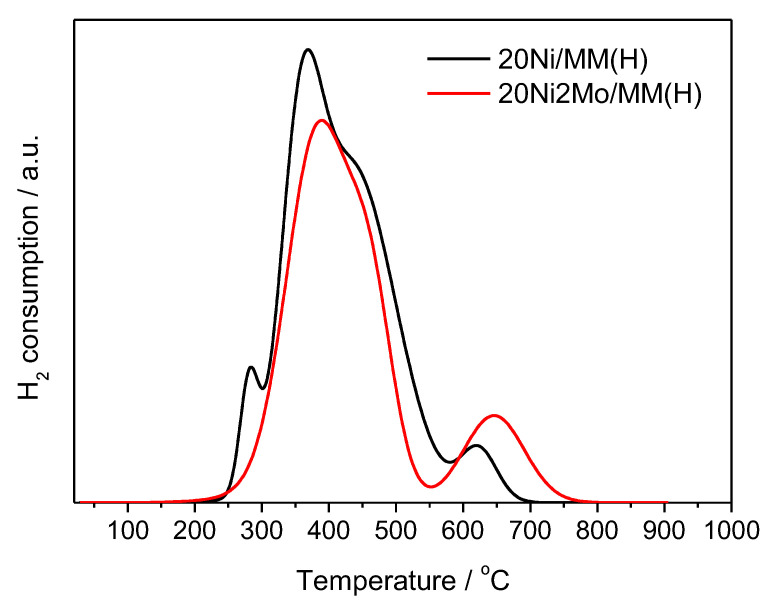
H_2_–TPR profiles of the 20Ni/MM(H) and 20Ni2Mo/MM(H) samples.

Figure 9 illustrates the H_2_–TPR curves recorded for the 20Ni/MM(H) and 20Ni2Mo/MM(H) samples. These indicate that the reduction temperature of 500 °C selected for the activation of the catalysts was sufficient for almost complete reduction of the supported Ni phase. An inspection of the curve corresponding to the unpromoted sample indicates at least four kinds of nickel species reduced at different temperatures. The first reduction peak at about 280 °C was attributed to the reduction of NiO interacting weakly with the support, indicating that a fraction of the nickel phase is not well dispersed on the support surface [83,84,85]. The second and third peaks at 370 and 435 °C were attributed to the reduction of NiO species exhibiting medium and strong interactions with the support surface, thus indicating high dispersion of the active phase [83,84,85]. The final peak at 620 °C could be attributed to the reduction of Ni^2+^ species incorporated in the support lattice, creating a mixed phase [83,84,85]. The aforementioned first reduction peak (280 °C) disappears in the H_2_–TPR curve of the promoted sample, indicating a favorable influence of the Mo promoter on the dispersion of the nickel active phase. This is also confirmed by the shift observed in the maxima of the second (370 → 390 °C) and third (435 → 440 °C) reduction peaks at higher temperatures. A similar shift was observed for the high-temperature peak, which was shifted from 620 (in the curve of the unpromoted sample) to 645 °C, probably indicating a strong interaction between the nickel and molybdenum phases.

**Figure 10:**
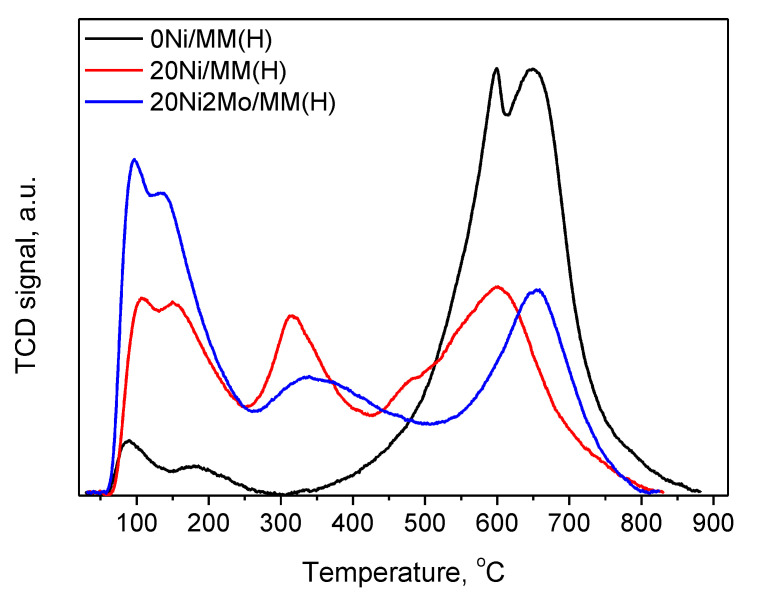
NH_3_-TPD profiles of 0Ni/MM(H), 20Ni/MM(H) and 20Ni2Mo/MM(H) samples.

Figure 10 illustrates the temperature-programmed desorption profiles of chemisorbed ammonia on 0Ni/MM(H), 20Ni/MM(H) and 20Ni2Mo/MM(H) catalysts. In this figure, three types of acid sites can be observed, developed on the catalyst surface. The first peak, centered at 100–200 °C, is assigned to the desorption of ammonia from Lewis acid sites, as the weak coordinate bonds break at low temperatures. The second and third peaks around 200–370 °C and 370–650 °C are due to the desorption of ammonia molecules from Brønsted intermediate and strong acid sites, respectively [86]. The deposition of the nickel phase on the support surface increased the weak and intermediate acidity, while it diminished the strong acidity of the support. This indicates that Ni^2+^ species are preferentially deposited on the strong Brønsted acid sites of the support, probably following an ion-exchange mechanism. The addition of the promoter (Mo species) further increased the weak acid sites of the corresponding catalyst.

**Figure 11:**
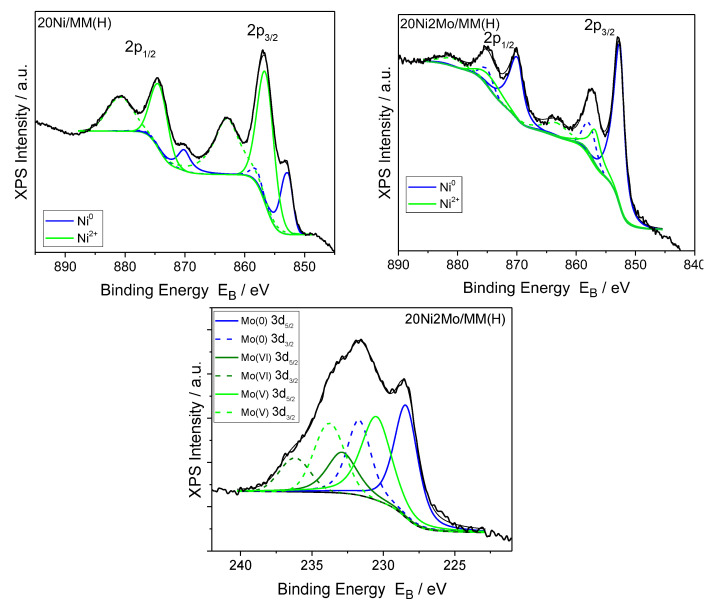
Deconvoluted XPS Ni2p and Mo3d peaks of the 20Ni/MM(H) and 20Ni2Mo/MM(H) catalysts.

Figure 11 indicates that the addition of the Mo promoter to a nickel catalyst supported on montmorillonite favors the reduction of the Ni phase, in accordance with the electron diffraction results (Figure 5). Regarding the Mo species, the XPS analysis of the Mo3d peaks revealed that most of the Mo species are in the oxidation states (VI) and (V), while only a few are in the metallic state.

### 2.2. Catalyst Evaluation

**Figure 12:**
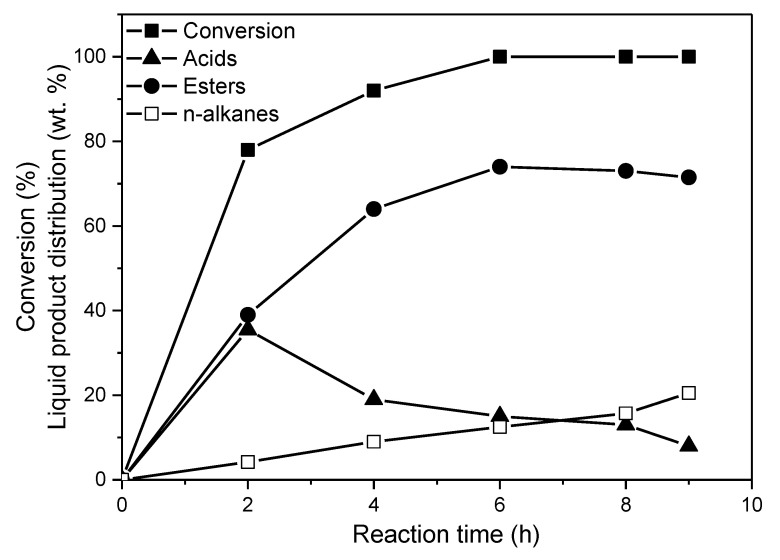
Conversion (%) of WCO achieved over the 20Ni2Mo/MM(H) catalyst and the liquid product distribution (wt.%) obtained at various reaction times (reaction conditions: 100 mL of WCO, $P_{H_{2}}$ = 40 bar, T = 310 °C and H_2_ flow = 100 mL/min).

Table 2 illustrates the evaluation parameters determined with regard to the SDO of WCO over the catalysts studied (reaction conditions: 100 mL of WCO, $P_{H_{2}}$ = 40 bar, T = 310 °C, H_2_ flow = 100 mL/min and reaction time = 9 h). It can be seen that the main products of the reaction are fatty acids, fatty acid–fatty alcohol esters and n-alkanes (C15, C16, mainly C17 and C18). The intermediates (fatty acids and fatty acid–fatty alcohol esters) and final products (n-alkanes) are compatible with the SDO network over the nickel catalysts described in detail in the Introduction (Figure 1). Moreover, the reaction kinetics curves (amount of a given product versus time) (Figure 12) pass through a maximum for the intermediate products, whereas they increase monotonically for the n-alkanes, in line with the well-established network over nickel catalysts (Figure 1) [11,43]. This clearly shows that this network is also valid for nickel catalysts supported on mineral montmorillonite.

**Figure 13:**
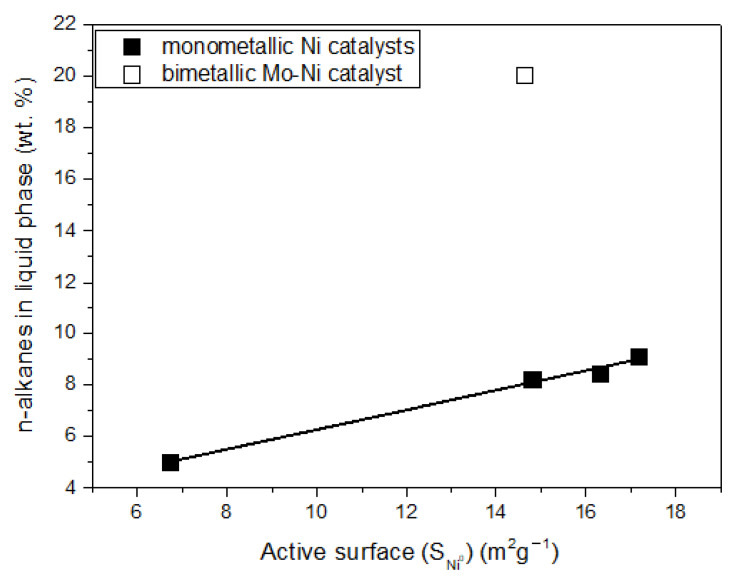
The n-alkanes concentration in the liquid product of the reaction vs. Ni active surface, over both monometallic and bimetallic catalysts.

The activity of the catalysts, determined by the % conversion of the WCO, increased with the nickel content and reached 100% over the catalyst containing 30 wt.% Ni. The catalyst efficiency, expressed as the % composition of the liquid phase in the n-alkanes, increased considerably with nickel content from 10 to 20 wt.% Ni and slightly for higher Ni contents, following the slight increase observed for the active surface in the range of nickel content of 20–40 wt.% (Table 1). Therefore, a close relation between the catalytic efficiency and active surface is anticipated, indicating that nickel is the active phase (Figure 13). The value of the ratio (C15 + C17)/(C16 + C18) was higher than unity in all cases, indicating that SDO mainly proceeded through the decarbonylation of the intermediate aldehydes (see Figure 1).

The above results indicated that a loading of 20 wt.% Ni was quite sufficient for a Ni catalyst supported on montmorillonite, for SDO of WCO under solvent-free conditions. Based on this finding and on the relevant literature [20,21,48,49,50,51,52,53,54,55,56,57,58,59,60,61,62,63], a catalyst containing 20 wt.% Ni and 2 wt.% Mo co-deposited on the montmorillonite surface was also synthesized and studied. In this catalyst the atomic ratio $\frac{Ni}{Ni+Mo}$ was equal to 0.94, which is considered very suitable according to the literature [20,21,49,50,52,55,56,59,62,63].

The presence of Mo species resulted in an increase in the % WCO conversion from 77.9% to 100% and a drastic increase in the amount of the n-alkanes from 8.2 to 20 wt.% (Table 2, Figure 13). Therefore, the promoting action of the Mo species was clearly demonstrated for the first time for nickel catalysts supported on montmorillonite. The increase in the amount of n-alkanes is the most important evaluation parameter, as it reflects the increase in the green diesel yield. Moreover, the presence of the Mo species resulted in a considerable decrease in the (C15 + C17)/(C16 + C18) ratio (Table 2), indicating that this favors the SDO pathway taking place through the dehydration of the intermediate alcohols, in agreement with the literature [20,21].

The promoting action of molybdenum could be partly attributed to the increase in the nickel dispersion implied by the H_2_-TPR profiles (Figure 9) and the increase in the reduction of the well-dispersed NiO (not detectable by XRD (Figure 4) but visible by electron diffraction (Figure 5)) deduced via XPS (Figure 11). However, taking into account the fact that the promoted and the corresponding unpromoted catalysts exhibited almost the same active surface (Table 1) and similar nickel particle size distributions (Figure 6), the molybdenum promoting action cannot be mainly attributed to structural Mo effects on the supported nickel nanoparticles. This can be inferred from the observation that the efficiency over the promoted catalyst was much higher than that expected on the basis of the active surface (Figure 13). Our results indicate that the support surface of the promoted catalyst is covered by well-dispersed metallic nickel nanoparticles (XRD, XPS) and very well-dispersed Mo(V) and Mo(Vi) molybdenum oxides (XPS), besides the metallic Mo nanoparticles. Moreover, the SEM–EDX mapping of the nickel and molybdenum showed that the nickel and molybdenum species are located very close together, at least at the microscale (Figure 8). Based on the above, and taking into account the relevant literature [20,49,53], it seems that the Mo promoting action can be mainly attributed to the synergy between the oxygen vacancies situated on the surface of the molybdenum oxides and the neighboring metallic nickel sites. The intermediate fatty acids are adsorbed on the oxygen vacancies through the hydroxylic oxygen of the C-OH group. Thus, the C-O bond is activated, facilitating its attack by hydrogen atoms formed through the dissociative adsorption of hydrogen molecules on nickel sites. This attack results in the transformation of the fatty acids to the corresponding fatty aldehydes. Consequently, the slowest step of the SDO network presented in the Introduction (Figure 1) is accelerated, justifying the Mo promotion. The aldehydes are also adsorbed on oxygen vacancies via the oxygen of the C=O bond, which is activated. This accelerates their reduction to the corresponding fatty alcohols, explaining the increasing participation of the dehydration pathway in the whole SDO network (Figure 1).

In an attempt to increase further the green diesel yield obtained over the 20Ni2Mo/MM(H) catalyst, various reaction parameters were investigated. A recently published and very systematic work on this subject [55] showed that increasing the reaction temperature in the range of 280–340 °C significantly increased the yield of hydrocarbons. Moreover, increasing the catalyst amount up to 5% results in an increase in the amount of hydrocarbons obtained in the liquid mixture. In addition, a residence time of about half an hour is enough for obtaining the maximum amount of hydrocarbons at the fairly high H_2_ pressure of 60 bar, in the presence of 5% catalyst.

**Figure 14:**
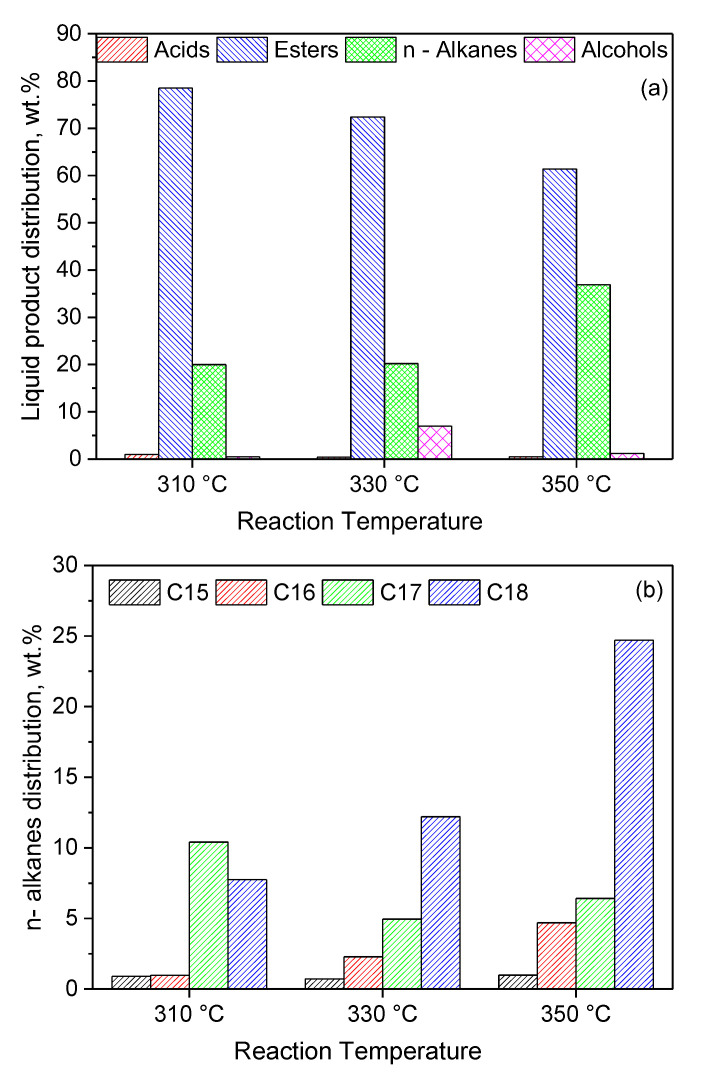
Composition of the liquid phase in n-alkanes, acids, alcohols and esters (**a**) and n–alkanes distribution (**b**), obtained for various reaction temperatures over 1 g of the 20Ni2Mo/MM(H) catalyst, after 9 h of reaction (reaction conditions: 100 mL of WCO, $P_{H_{2}}$ = 40 bar, H_2_ flow = 100 mL/min).

In view of the above, we firstly performed catalytic tests at higher reaction temperatures (330 and 350 °C). Figure 14a shows that an increase in the reaction temperature resulted in a decrease in the amount of intermediate esters and a considerable increase in the amount of green diesel hydrocarbons (C15, C16, C17 and C18) from 20 to 37%. Taking into account the fact that the esterification reactions are endothermic [87], one may conclude that the diminution in the amount of esters, as the temperature increases, is not due to the effect of temperature on the reaction equilibrium but on the kinetics of the relevant reactions. A small amount of the intermediate alcohols was identified at 330 °C, in agreement with the SDO mechanism described in the Introduction (Figure 1). Inspection of Figure 14b shows that an increase in the reaction temperature favors the production of hydrocarbons with an even number of carbon atoms, implying a shift of the SDO pathway from the decarbonylation of the intermediate aldehydes to the dehydration of the intermediate alcohols. Moreover, this can be related to the decrease in the amount of esters with increasing reaction temperature, as the SDO of the intermediate esters favors the production of hydrocarbons with even numbers of carbon atoms (see the SDO network in Figure 1 and [22,43]). The production of C18 and C16 hydrocarbons instead of C17 and C15 hydrocarbons is obviously beneficial from the viewpoint of carbon atom economy.

At this point, it should also be mentioned that a very small amount of cracking products (<1%) was detected in the liquid reaction product, and only in the case of the 20Ni2Mo/MM(H) catalyst working at the high temperature of 350 °C.

**Figure 15:**
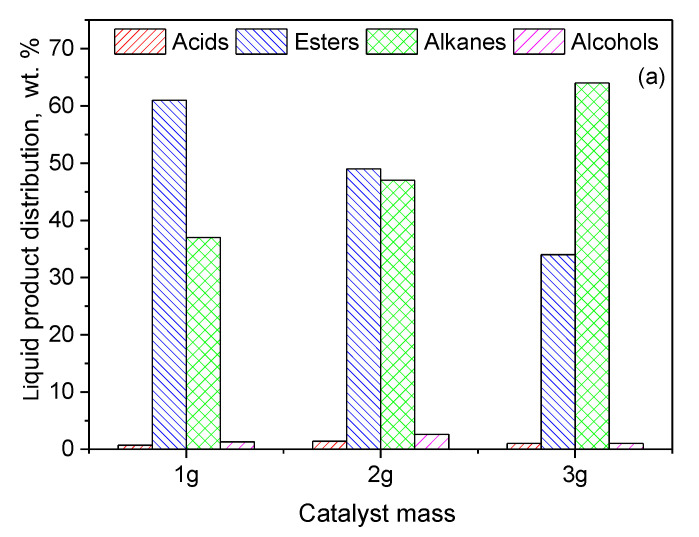

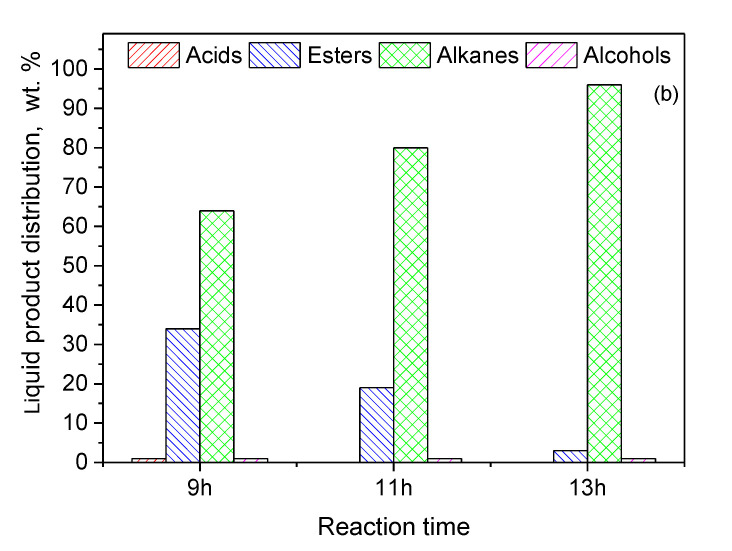
Composition of the liquid phase in n-alkanes, acids, alcohols and esters obtained for (**a**) various catalyst masses of the 20Ni2Mo/MM(H) catalyst at 350 °C after 9 h and (**b**) various reaction times over 3 g of the 20Ni2Mo/MM(H) catalyst at 350 °C (reaction conditions: 100 mL of WCO, $P_{H_{2}}$ = 40 bar, H_2_ flow = 100 mL/min).

In order to increase even further the green diesel yield obtained over the 20Ni2Mo/MM(H) catalyst, catalytic tests were performed at 350 °C with three different values of the catalyst mass. As expected, an increase in the catalyst mass caused an increase in the amount of green diesel hydrocarbons from 37 to 64% (Figure 15a).

Finally, we investigated the effect of reaction time on the catalytic efficiency. The results are illustrated in Figure 15b. It can be seen that an almost full transformation of the WCO to green diesel (96%) was obtained over the promoted catalyst at 350 °C, for a reaction time of 13 h and a ratio of WCO volume to catalyst mass of 100 mL/3 g. This result shows the very high efficiency of the promoted catalyst, taking into account the fact that the latter two parameters correspond to an LHSV value equal to 2.5 h^−1^, which is considered quite a reliable value from the viewpoint of industrial applications.

The comparison of the present results with current literature results in the domain of the SDO of triglycerides and related compounds over nickel catalysts clearly revealed that the catalytic behavior of nickel species supported on mineral montmorillonite was similar to that exhibited over synthetic supports such as γ-alumina [20,21,49,50,51,63], silica [52,53,56,57,60], zirconia [88], ceria [64], zeolites [54,58,59,63], active carbons [62,63] and mineral palygorskite [66,67], from the viewpoint of the SDO network. Moreover, the catalytic efficiency obtained in the present study over nickel species supported on post-treated mineral montmorillonite was comparable to those obtained over nickel species supported on synthetic supports, though a rigorous comparison is very difficult to achieve due to the quite different evaluation conditions adopted in each case. Moreover, the comparison of the present results regarding the Mo–Ni bimetallic catalyst with the corresponding catalysts supported on various synthetic supports [20,21,49,50,51,52,53,54,55,56,57,58,59,60,61,62,63,64,65] showed that the Mo promoting action was also clearly manifested over nickel catalysts supported on mineral montmorillonite.

## 3. Materials and Methods

Following the approach adopted in this study, as outlined in the Introduction, the experimental part of the work involved the following steps. (i) The acid treatment with 2N HCl aqueous solution of the mineral montmorillonite to improve its textural characteristics. This treatment had been previously tested successfully [75]. (ii) The preparation of supported nickel catalysts with various nickel loadings in the range 10–40 wt.%, which had been proved very suitable in the case of nickel catalysts supported on mineral palygorskite [66,67]. (iii) The evaluation of these catalysts for selecting the most promising loading. (iv) The preparation of a molybdenum-promoted nickel catalyst based on the most promising loading of the unpromoted nickel catalyst, synthesized with the optimum atomic Ni/(Mo+Ni) ratio [20,21]. (v) The evaluation of the Mo–Ni catalyst that had proved to be the most active in the temperature range 310–350 °C, which is considered appropriate for SDO [11] (the upper limit of this range is critical because at higher temperatures extended fragmentation of the triglyceride side chains is probable). (vi) The evaluation of the Mo–Ni catalyst at the most promising reaction temperature (350 °C) for different reaction times and volume of WCO/mass of catalyst ratios, in order to optimize the catalytic efficiency. (vii) The characterization of the catalysts using various methods in order to rationalize the catalytic efficiency at each step of the study.

### 3.1. Feedstock

The WCO was purchased from the CollectOil company (Patras, Greece). It was filtered several times using filters of different sizes and then centrifuged. The feedstock characteristics have been reported in a previous contribution [89].

### 3.2. Catalyst Preparation

#### 3.2.1. Acid Treatment of Mineral Montmorillonite

The mineral montmorillonite (MM), originating from soils of the Greek island Milos in the Aegean Sea, was provided by the Imerys company (Paris, France). This was treated with 2N HCl aqueous solution in order to improve its physicochemical properties. In a typical experiment, 5 g of the montmorillonite and 100 mL of the acid solution were added to a round-bottomed flask. The acid treatment was performed at 70 °C for 6 h. The suspension was cooled at room temperature and then filtered under vacuum using a Büchner funnel sintered disc with a porosity of 2 (40–90 μm). The solid in the filter was washed several times with distilled water in order to remove the acid remaining inside its pores. The wet solid obtained was dried overnight at 110 °C and then ground to a fine-grained texture. More details of the acid treatment of mineral montmorillonite have been reported elsewhere [75].

#### 3.2.2. Synthesis of the Catalysts

Monometallic Ni catalysts supported on montmorillonite were synthesized following the deposition–precipitation method. A suitable amount of nickel nitrate hexahydrate (Chem-Lab NV) was added to a spherical flask containing 50 mL of water with a weighed amount of montmorillonite suspended in it. Then, 50 mL of urea (Duchefa) in aqueous solution was added. The concentration of the solution was adjusted to obtain a molecular ratio of ammonia/NO_3_^−^ ions equal to 3. After the installation of a reflux condenser and a magnetic stirrer, the flask was immersed in a heated oil bath (80 °C), and the suspension remained at this temperature for 10 h. The temperature was then decreased to room temperature and the suspension was filtered. The solid obtained was washed with triply distilled water and dried at 110 °C overnight. The MoNi bimetallic catalyst was synthesized following a co-deposition—precipitation methodology involving the dissolution of suitable amounts of nickel nitrate hexahydrate and ammonium heptamolybdate tetrahydrate (Alfa Aesar) in a spherical flask containing 50 mL of water. The rest of the procedure was identical to that described above.

#### 3.2.3. Activation of the Catalysts

The activation procedure, performed in a fixed-bed quartz reactor, involved two steps. In the first step, carried out under a flow of argon (30 mL/min), the temperature was increased at a rate of 5 °C/min from room temperature up to 400 °C and then held constant for 1 h. The nickel hydroxide, formed via deposition–precipitation, was decomposed to nickel oxide in this step (oxidic precursors). The activated catalysts were obtained in the second step by reducing the oxidic precursors at 500 °C for 2.5 h under a flow of hydrogen (30 mL/min).

### 3.3. Catalyst Characterization

#### 3.3.1. N_2_ Adsorption—Desorption Isotherms

The BET specific surface area (S_BET_) and the pore volume distribution (PVDBJH) of the activated samples were determined using N_2_ adsorption—desorption isotherms recorded on a Micromeritics apparatus (TriStar 3000 porosimeter) (Norcross, GA, USA). The PVDBJH was determined following the BJH methodology based on the N_2_ desorption curve.

#### 3.3.2. X-ray Diffraction

The crystal phases and the crystal sizes of the activated samples were determined via XRD. The X-ray diffraction patterns were recorded in the 2θ range of 5–80° using a Bruker D8 Advance diffractometer. This was equipped with a Ni-filtered CuKa (1.5418 Å) radiation source (step size 0.02°, time per step 0.5 s) (Karlsruhe, Germany).

#### 3.3.3. Scanning and Transmission Electron Microscopy

A scanning electron microscope (JEOL JSM-6300) (Peabody, MA, USA) equipped with an energy-dispersive spectrometer (EDS) was used for determining the morphology and the elemental mapping in the activated samples. A JEOL JEM-2100 system operating at 200 kV (resolution: point 0.23 nm, lattice 0.14 nm) was used for obtaining the TEM images and electron diffraction data. The TEM images were recorded using an Erlangshen CCD Camera (Gatan Model 782 ES500W, Pleasanton, CA, USA).

#### 3.3.4. Hydrogen Temperature Programmed Reduction

The H_2_-TPR experiments were performed in order to investigate the reducibility of two precursor (oxidic) samples. To record the H_2_-TPR curves, a quantity of the sample, taken before activation, was placed in a quartz micro-reactor, and a H_2_–He mixture was passed through the reactor for a certain time at room temperature. The temperature was then increased to 1000 °C at a constant rate of 10 °C/min. The decrease in hydrogen concentration in the gas mixture due to the reduction of the sample was monitored using a TCD. The gas mixture in the reactor outlet was dried in a cold trap placed before the TCD.

#### 3.3.5. Ammonia Temperature Programmed Desorption

The NH_3_-TPD experiments were carried out in order to determine the distribution of the surface acid sites developed on two activated samples. A certain quantity of the solid was placed in a quartz micro-reactor and He was passed through to remove the adsorbed species. A stream of NH_3_ was fed in at room temperature. Then, this was switched to He to remove the physisorbed NH_3_. The temperature was then increased up to 900 °C at a rate of 10 °C/min, and the amount of desorbed NH_3_ was determined at the reactor outlet using a TCD.

#### 3.3.6. X-ray Photoelectron Spectroscopy

The photoemission experiments were carried out in an ultra-high vacuum system (UHV) consisting of a high-pressure cell and preparation and analysis chambers. The base pressure in the analysis chamber was 1 × 10^−9^ mbar. The analysis chamber was equipped with a dual-anode Mg/Al X-ray gun and a SPECS Phoibos 100 1D-DLD energy analyzer. A monochromatized MgKα line at 1253.6 eV and an analyzer pass energy of 15 eV were used in the XPS measurements, giving a full width at half maximum (FWHM) value of 0.9 eV for the Ag3d5/2 peak. The XPS core level spectra were collected and analyzed using SpecsLab Prodigy commercial software (version 4.86.2) (SPECS GmbH, Berlin). The atomic ratios were calculated from the intensities (peak areas) of the XPS peaks weighted with the corresponding relative sensitivity factors (RSF). The samples were in powder form and pressed into pellets (stainless steel). Prior to XPS analysis, the samples were heated at 500 °C in a H_2_ atmosphere for 5 h in the high-pressure cell.

### 3.4. Catalyst Evaluation

The catalyst efficiencies for the transformation of WCO into third-generation green diesel were evaluated in a semi-batch reactor (300 mL, Autoclave Engineers, Erie, PA, USA) working at various temperatures (310–350 °C) at a hydrogen pressure of 40 bar. The reactor was loaded with a feedstock volume of 100 mL and a catalyst mass of 1–3 g. Then, it was heated at a temperature rate of 10 °C/min to the desired reaction temperature under an Ar flow (100 mL/min) to purge the dead volume from the ambient air. When the desired reaction temperature was achieved, the Ar stream was changed to H_2_ at the same flow rate, controlled by a mass flow controller (Brooks 58505 S, Smethwick, UK). The reaction was monitored for 9 h. The best catalyst was evaluated for up to 13 h. At the exit of the reactor, a cold trap was used to collect water and organic products evaporated under the reaction conditions. Liquid samples were withdrawn from the reactor every hour and the reaction products were determined via gas chromatography. A Shimadzu GC-2010 Plus gas chromatograph was used, equipped with a flame ionization detector and a suitable column (ZB-5HT Inferno, Zebron, l: 30 m, d: 0.32 mm, tf: 0.10 μm). The reaction products were also identified via gas chromatography–mass spectrometry using a Shimadzu GCMS-QP2010 Ultra apparatus.

## 4. Conclusions

The catalytic efficiency increased significantly with nickel content from 10 to 20 wt.% Ni, and then slightly from 20 to 40 wt.% Ni, following the trend of increase observed for the nickel active surface. The addition of 2 wt.% Mo to the catalyst containing 20 wt.% Ni resulted in a considerable increase in the amount of green diesel hydrocarbons in the reaction product from 8.2 to 20%. Thus, the Mo promoting action was demonstrated for nickel catalysts supported on montmorillonite. The Mo species, moreover, led to a considerable decrease in the (C15 + C17)/(C16 + C18) ratio, indicating that Mo favors the SDO pathway taking place through the dehydration of the intermediate alcohols. The promoting action of Mo was attributed mainly to the synergy between the oxygen vacancies situated on the surface of the very well-dispersed Mo(V) and Mo(VI) oxides and the neighboring metallic nickel sites, and only secondarily to the structural Mo effects on the supported nickel nanoparticles. An increase in the reaction temperature from 310 to 350 °C caused an additional increase in the green diesel hydrocarbons in the reaction product from 20 to 37% and a drastic decrease in the (C15 + C17)/(C16 + C18) ratio from 1.3 to 0.25. The latter is beneficial from the viewpoint of carbon atom economy. Furthermore, an increase in catalyst mass from 1 to 3 g per 100 mL of WCO resulted in a drastic increase in green diesel hydrocarbons in the reaction products from 37 to 64%. Finally, an increase in the reaction time from 9 to 13 h led to an almost full transformation of the WCO to green diesel (96%). This result is very important, considering the solvent-free reaction conditions as well as the fairly high WCO-volume-to-catalyst-mass ratio (33.3 mL/g) and the reaction time of 13 h, which correspond to a LHSV equal to 2.5 h^−1^. This value is considered to be quite reliable from the viewpoint of industrial applications. Thus, the cheap and abundant mineral montmorillonite is a promising support for developing efficient nickel—molybdenum catalysts for third-generation green diesel production.

## Figures and Tables

**Table 1:** Physicochemical characteristics of the supports and the catalysts.

Sample	S_BET_ (m^2^g^−1^)	S_micro_ (m^2^g^−1^)	S_BJH_ (m^2^g^−1^)	S_Ni_^0^ (m^2^g^−1^) ^1^	PV (cm^3^g^−1^)	MPD (nm)	MCS_Ni_^0^ (nm)
MM	62	19	43	-	0.16	9.3	-
MM(H)	136	22	114	-	0.22	5.6	-
0Ni/MM(H)	48	12	36	-	0.15	9.7	-
10Ni/MM(H)	62	19	43	6.7	0.14	7.4	10.0
20Ni/MM(H)	90	36	54	14.8	0.15	7.5	9.1
30Ni/MM(H)	104	40	64	16.3	0.15	7.9	12.4
40Ni/MM(H)	83	29	54	17.2	0.14	5.8	15.7
20Ni2Mo/MM(H)	100	15	85	14.7	0.18	6.4	9.2

^1^ S_Ni_^0^: specific surface area per gram of catalyst exposed by the supported nickel nanoparticles (active surface) calculated from XRD data on the mean crystal size of metallic nickel (MCS_Ni_^0^) in the catalysts.

**Table 2:** Conversion of WCO (% X_WCO_) and composition of the liquid mixture obtained upon SDO of WCO at 310 °C for 9 h, over the catalysts studied.

Sample	X_WCO_ %	Acids (wt.%)	Esters (wt.%)	n-Alkanes (wt.%)	(C15 + C17)/(C16 + C18)
10Ni/MM(H)	73.8	27.3	41.5	5	2.2
20Ni/MM(H)	77.9	26.7	43.0	8.2	2.2
30Ni/MM(H)	100	3.9	87.7	8.4	1.9
40Ni/MM(H)	100	3.5	87.4	9.1	1.8
20Ni2Mo/MM(H)	100	1	78.5	20	1.3

## Data Availability

Not applicable.

## Sample Availability

Samples of all catalysts are available from the authors.
